# The role of heat shock proteins in preventing amyloid toxicity

**DOI:** 10.3389/fmolb.2022.1045616

**Published:** 2022-12-15

**Authors:** Ricarda Törner, Tatsiana Kupreichyk, Wolfgang Hoyer, Jerome Boisbouvier

**Affiliations:** ^1^ University Grenoble Alpes, CNRS CEA Institut de Biologie Structurale (IBS), Grenoble, France; ^2^ Institute of Biological Information Processing (IBI-7: Structural Biochemistry), JuStruct: Jülich Center for Structural Biology, Forschungszentrum Jülich, Jülich, Germany; ^3^ Institut für Physikalische Biologie, Heinrich-Heine-Universität Düsseldorf, Düsseldorf, Germany

**Keywords:** chaperones, amyloid, structural biology, HSP, aggregation

## Abstract

The oligomerization of monomeric proteins into large, elongated, β-sheet-rich fibril structures (amyloid), which results in toxicity to impacted cells, is highly correlated to increased age. The concomitant decrease of the quality control system, composed of chaperones, ubiquitin-proteasome system and autophagy-lysosomal pathway, has been shown to play an important role in disease development. In the last years an increasing number of studies has been published which focus on chaperones, modulators of protein conformational states, and their effects on preventing amyloid toxicity. Here, we give a comprehensive overview of the current understanding of chaperones and amyloidogenic proteins and summarize the advances made in elucidating the impact of these two classes of proteins on each other, whilst also highlighting challenges and remaining open questions. The focus of this review is on structural and mechanistic studies and its aim is to bring novices of this field “up to speed” by providing insight into all the relevant processes and presenting seminal structural and functional investigations.

## 1 Introduction

To date, about 50 proteins and peptides have been identified to be implicated in amyloid diseases (Iadanza et al., 2018). The hallmark of these pathologies is the aggregation of small monomeric proteins into large elongated fibril structures (amyloid), which results in death of the affected cells. Most studied and known are neurodegenerative diseases, such as Alzheimer’s or Parkinson’s disease, but the formation of aberrant fibrillar aggregates can also be systemic or localized in peripheral organs (Cho et al., 2018). What unites the large majority of these diseases is that no treatment is available yet, although they have received extensive attention from the scientific community (Chiti & Dobson, 2017). The main risk factor for the development of these diseases is age, therefore they increasingly become a threat in our long-lived societies. Although the presence of amyloid aggregates is clearly related to disease, the link is not straightforward. Quantity of aggregates does not correlate well with severity of disease and disaggregation of fibrils does not alleviate symptoms (Espay et al., 2019). So, in order to address amyloid diseases therapeutically, a detailed molecular understanding of the pathological process is necessary. Although major progress has been made (Chiti & Dobson, 2017) in the years since the term amyloid was coined in 1854 (Sipe & Cohen, 2000), much remains to be elucidated about the mechanism of pathology.

Chaperones have been shown to intervene in the fibrillation process of amyloidogenic proteins, during multiple steps, by inhibition of aggregation, disaggregation or detoxification of already formed amyloid fibrils (A. Wentink et al., 2019). In the following, we summarize how chaperone molecules, as modulators of protein conformational states, are able to act to prevent amyloid toxicity. Both amyloidogenic proteins and chaperones are introduced and the major biological processes are discussed before the special nature of their interaction is examined. Lastly, we present examples of relevant structural studies of heat shock proteins (HSPs), the major class of molecular chaperones, and their interaction with amyloidogenic proteins and analyze the implications of the findings. The mechanistic study of these interactions allows to gather important insights and helps to shape the path for future use of chaperones to address currently incurable amyloidogenic diseases.

## 2 Amyloidogenic proteins

### 2.1 The universality of amyloid structure

**FIGURE 1:**
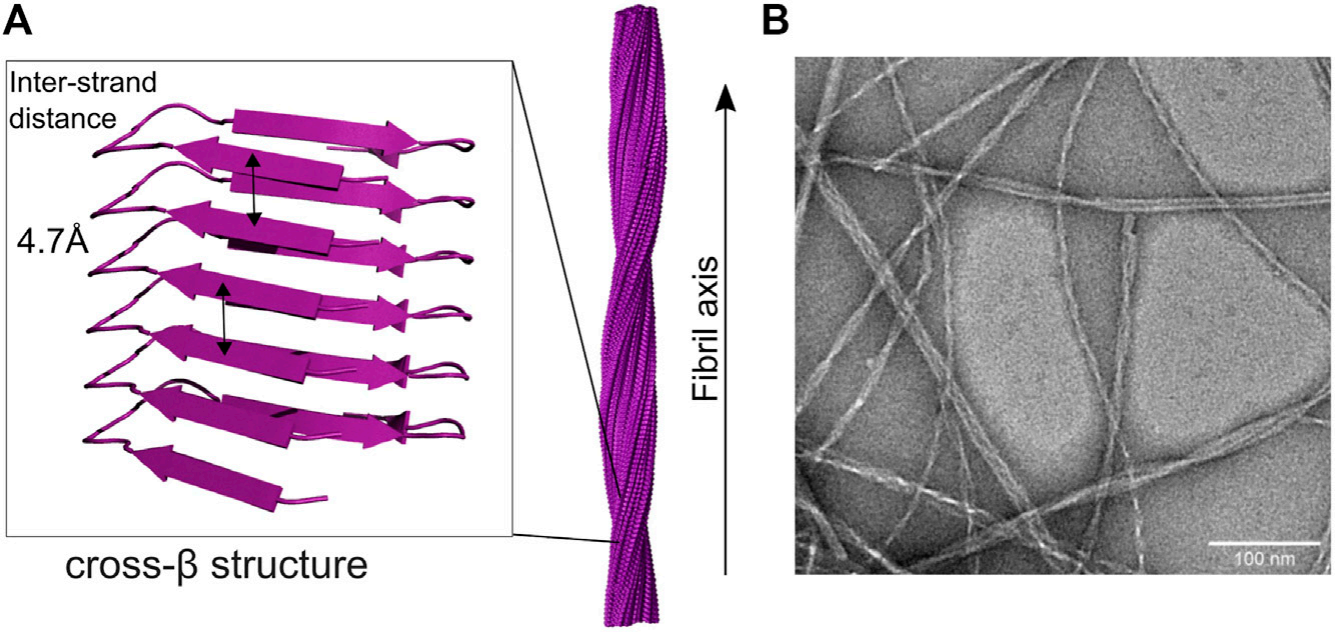
Cross-beta and fibril structure of amyloid fibrils **(A)** in the fibril, monomers form beta-sheet structures along the long axis of the fibril, with beta-strands arranged perpendicular to the fibril axis. The characteristic spacing of the sheets gives rise to typical diffraction peaks, hence cross-beta structure. Multiple protofilaments twist around each other, thereby forming the elongated fibril structure [here seen for IAPP (Röder et al., 2020)] **(B)** The twisted, elongated fibril structure can be seen under the electron microscope, here for IAPP fibrils. Note the polymorphism of the observed fibrils.

Amyloid is the result of aggregation of small monomeric proteins into large elongated fibril structures. Amyloid fibrils are not unstructured aggregates, but a highly regular arrangement of monomers into a fibril structure which can be microns in length. The proteins constituting the monomeric building blocks form β-sheets, which are arranged perpendicular to the fibril axis (Figure 1A) (Sunde et al., 1997). This structure element is the defining characteristic of amyloid fibrils and can be observed in X-ray diffraction experiments as “cross-β” structure (Geddes et al., 1968) or detected by interaction with specific dyes, such as Thioflavin T (ThT) or Congo Red, which arrange themselves in specific ways on the fibril surface (Gade Malmos et al., 2017). The name amyloid stems from this interaction with dyes, which also color starch (amylon). Structure elucidation has been slow, as fibrils cannot be crystallized due to their inherent twist and are non-soluble (Landreh et al., 2016). So for a long time, the only structural information came from crystallization of small fragments and solid state-NMR studies (SS-NMR) (Lührs et al., 2005; Streltsov et al., 2011), but with the advent of the resolution revolution in cryo-electron microscopy (cryo-EM) (Callaway, 2015) the number of elucidated fibril structures has been growing, and multiple structures of all major disease related proteins are available now (Gremer et al., 2017; Guerrero-Ferreira et al., 2018; Kollmer et al., 2019; Cao et al., 2020; Gallardo et al., 2020; Röder et al., 2020). The natural twist of the β-sheet structure element translates to a twist in the fibril. Typically, the full fibril is composed of multiple protofilaments (most commonly two), twisting around each other (Figure 1B), but also single protofilament structures have been reported. Within each β-sheet, the neighboring β-strands form hydrogen bonds between each other along the fibril axis, which gives the fibril mechanical strength and stability (Knowles et al., 2007). This generic cross-β structure element allows polymorphism and it is frequently observed that the same peptide is able to form different fibril structures, both in the molecular structure of the protofilaments as well as in the number and arrangement of protofilaments (Cao et al., 2020; Gallardo et al., 2020; Röder et al., 2020). However, usually the presence of a given polymorph is able to seed the same structure, even under different growth conditions (Petkova et al., 2005). As the *ß*-sheet interaction is mediated by the peptide backbone, multiple arrangements of sidechains are possible and are indeed established, possibly due to the absence of evolutionary selection converging towards a single structure (Chiti & Dobson, 2017). Even so, there is some prevalence regarding the arrangement of *ß*-sheets. Typically, *ß*-strands are in register, meaning all strands packing on top of each other are oriented in the same direction and identical amino acid sequence segments stack precisely on top of one another, forming a parallel *ß* -sheet. The interaction between *ß*-sheets within protofilaments or at the protofilament interface is mediated *via* interdigitation of sidechains in a steric zipper motif, which leads to exclusion of water molecules on the interface (Landreh et al., 2016).

### 2.2 Similarities between amyloidogenic proteins and prediction of amyloidogenicity

It has been proposed that the formation of cross-β structure, as observed in amyloid aggregates, is a common property of peptide chains (Chiti & Dobson, 2006). This was initially concluded from the observations that many proteins which are not associated with diseases can be converted into the amyloid fold (In;aki Guijarro et al., 1998) and that proteins with polypeptide sequences composed of only one type of amino acid can fold into the amyloid state, (Fändrich & Dobson, 2002), thereby suggesting structural independence from the primary sequence. As the *ß*-fold is stabilized by backbone interactions, this generalization seems to hold. Yet, *in vivo* only a small subset of proteins, of about 50 proteins and peptides are observed to form amyloid structures, leading to amyloid-associated diseases. Chiti and Dobson (2017) have described certain regularities within this class of amyloidogenic proteins.

(1) Size: Whereas the average length of proteins encoded in the genome is 500 residues, the majority of amyloidogenic proteins have less than 400 residues, half of them even have fewer than 100 residues.
(2) Location: Protein aggregates are found in the compartments where the native protein typically resides. The majority of peptides and proteins found to form amyloid deposits are secreted, therefore forming deposits in the extracellular space. Only four peptides (α-synuclein, huntingtin, Tau, galectin 7) are cytosolic, forming intracellular inclusions.
(3) Heredity. Both hereditary and sporadic conditions fall under the category of protein misfolding diseases, only a few are transmissible. Hereditary cases generally have an early age of onset. They also tend to be autosomal dominant, leading to disease onset already if only one copy of the gene is impacted (Chiti & Dobson, 2017).

In order to predict aggregation probability on a molecular basis, multiple algorithms have been developed (DuBay et al., 2004; Fernandez-Escamilla et al., 2004; Pawar et al., 2005; Conchillo-Solé et al., 2007). It has been found that the physicochemical properties: hydrophobicity, charge, secondary structure propensity, polar and non-polar water-accessible surface areas, dipole moment, and stacking interaction of aromatic residues are important factors. That is, proteins with more hydrophobic surface exposed, lower charge, more aromatic residues, and stronger *ß*-sheet propensity tend to have higher aggregation rates.

Although a large part of amyloidogenic proteins are intrinsically disordered in their non-aggregated state, the analysis of these physicochemical factors leads to the finding that intrinsically disordered proteins have a lower aggregation propensity than globular proteins (Linding et al., 2004). Intrinsically disordered proteins contain less hydrophobic residues, more charges and of course little structural propensity. However, to adequately interpret these predictions one has to consider that these computations calculate aggregation propensity starting from an unfolded state, in which the typical folded protein spends only a fraction of its lifetime, whereas hydrophobic regions of intrinsically disordered proteins are basically constantly available for the creation of inter-molecular bonds.

An additional factor which is not amenable to prediction of amyloidogenicity by physicochemical properties are specific mutations which destabilize a native folded state. Also, it is clear that different sites have varying relative importance for the aggregation process, with the regions directly involved in the aggregation process being decisive (Chiti et al., 2002). Yet, the universality of the amyloid fold is not challenged by this as within these regions, aggregation propensity remains correlated with the aforementioned physicochemical factors and is not mediated *via* specific interactions.

### 2.3 Toxicity and the case of the structurally elusive oligomer

Despite the growing understanding of amyloid structure, to this day a sufficient proof of causal connection between toxicity and amyloid aggregates remains missing. It has become increasingly clear that mature fibrils are not as toxic as initially thought. For example, it has been found that the number of amyloid aggregates correlates poorly with disease severity (Espay et al., 2019). Although mature fibrils are not off the hook, as they play important catalytic roles during the fibrillation process and can be toxic by sequestration of proteins of the cell machinery (Olzscha et al., 2011; Labbadia & Morimoto, 2015), attention has shifted to other species emerging during the fibrillation process. In the context of amyloid formation, oligomers are usually defined as transiently formed assemblies of a relatively small number of protein molecules. Their structural elucidation is complicated by their short lifetime and/or by their lower structural order compared to the highly regular amyloid fibrils. Nevertheless, some specific oligomer structures were proposed, such as a *ß*-barrel with an antiparallel out-of-register *ß*-sheet core (Laganowsky et al., 2012). Many studies have focused on the Aβ peptide and reported oligomers of different size and shape both *in vitro* and *vivo*. (Teplow, 1998; Caughey & Lansbury, 2003). Early forming curvilinear, annular and spherical oligomers have been collectively termed protofibrils, as they occur before any significant amounts of amyloid fibrils are formed but are depleted once amyloid fibril formation takes over (Harper et al., 1997). As a note of caution, protofibrils should not be confused with protofilaments: Protofilaments are the cross-β structure stacks of protein molecules within amyloid fibrils, whereas protofibrils are transient, i.e., metastable, assemblies that differ structurally from amyloid fibrils. Metastable Aβ oligomers have also been denoted ADDLs or AβO. These metastable Aβ species are likely related; e.g., curvilinear protofibrils seem to be bead-on-a-string assemblies of multiple spherical ADDLs/AβOs (Chromy et al., 2003). Protofibrils are narrower than mature fibrils and up to 150 nm in length (Harper et al., 1997). While they also contain extensive *ß*-sheet structure, substantial evidence now indicates that they form in a reaction distinct from amyloid fibril formation, i.e., protofibrils are off-pathway with respect to amyloid fibril formation (Hasecke et al., 2018, 2021). This is supported by the observation that increased protofibril formation decreases the rate of amyloid fibril formation (Hasecke et al., 2018, 2021). Replacement of protofibrils by amyloid fibril occurs by dissolution of the protofibrils into monomers, followed by incorporation of the monomers into amyloid fibrils. Metastable Aβ oligomers are toxic to neuronal cell cultures and cause cognitive decline in animal models, with several detrimental AβO activities reported (Lambert et al., 1998; Cline et al., 2018). Current clinical trials suggest that targeting Aβ protofibrils is a viable approach to Alzheimer’s disease immunotherapy (Söllvander et al., 2018; Swanson et al., 2021).

It has been found that the exposure of hydrophobic residues on the oligomer surface is a major toxicity inducing factor (Ladiwala et al., 2012). Hydrophobic residues mediate unspecific interactions, so that through their exposure, undesirable interactions with components of the cell can occur. Increase in cell membrane permeability was found to play an important role in oligomer toxicity (Demuro et al., 2005). Model membrane studies have shown that many amyloid intermediates render bilayers permeable, both to small species such as ions but some even to large dyes (Arispe et al., 1994; HAI et al., 2001; Quist et al., 2005). Neither monomers nor mature fibrils are able to cause this effect (Hebda & Miranker, 2009). Transmembrane pores have not been experimentally proven (Lashuel & Lansbury, 2006), but multiple possible structures of transmembrane pores, both of α-helical or *ß*-barrel conformation are discussed (Zhao et al., 2014; Di Scala et al., 2016). However, increase of permeability does not necessarily have to be mediated by pores. Via their amphipathicity, amyloids can insert themselves into membranes, but also layer on top of membranes or remove lipid components *via* a detergent-like mechanism. Carpeting is binding of prefibrillar states to one leaf of a bilayer, thereby creating asymmetric pressure between the two leaves of the bilayer, which leads to increased permeability when the pressure is relaxed. Detergent effects are when protein is acting as a surfactant, removing lipid from the membrane and thereby thinning the membrane or creating a hole (Hebda & Miranker, 2009).

Another important factor for oligomer toxicity is size. Very small oligomers composed of two to three monomers and oligomers of more than 100 kDa are found to be not as toxic as the ones in the sweet spot in between (Mannini et al., 2012; Chiti & Dobson, 2017). Small oligomers can diffuse further through the cell and are therefore able to produce more damage. It is proposed that trafficking of oligomers between cells is an important mechanism by which amyloid diseases spread inside an organism (Brettschneider et al., 2015).

### 2.4 Kinetics of the aggregation process

**FIGURE 2:**
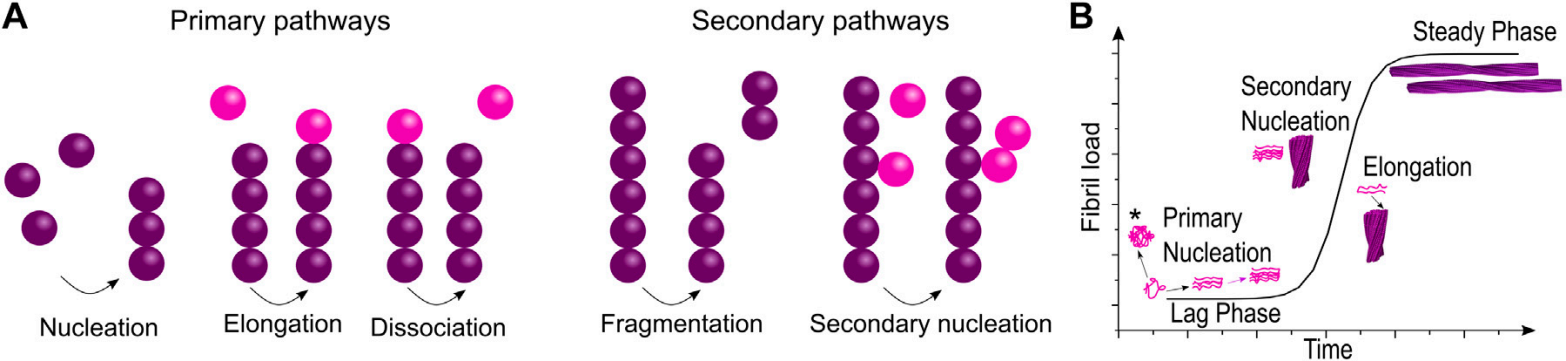
Model of fibrillation pathways at the molecular level and the macroscopically observed fibrillation kinetics. **(A)** The interplay of multiple microscopic processes determines the fibrillation process giving rise to **(B)** the macroscopic fibrillation curve. Although no part of the fibrillation curve corresponds purely to just one process, the influence of different fibrillation pathways can be extracted by fitting (*refers to off-pathway oligomers).

The importance of intermediate species in the fibrillation process is indisputable. Whilst structural studies of oligomers have been increasing only in recent years, the kinetics of fibril formation has been studied for decades and on this basis models describing the fibrillation process at a molecular level have been developed. Similar to the related process of crystallization, the aggregation process is described as a nucleation-dependent assembly. Aggregation starts with a stochastic oligomerization event, where a nucleus is formed, which then rapidly elongates by templated incorporation of monomers. A nucleus is defined as the smallest species that is able to initiate fibril elongation, that is, the rate of monomer addition is higher than the rate of monomer release; no specific structure is associated with this term. As soon as nucleation has occurred, the aggregate growth rate increases. The typically very strong exponential behavior of amyloid formation kinetics is an indicator for the occurrence of secondary processes, which keep on increasing the rate of the fibrillation, even though the monomer concentration is increasingly depleted. Secondary processes found to be a part of the fibrillation process in particular are fibril fragmentation and secondary nucleation (Figure 2A) (Morris et al., 2009). Both processes lead to the formation of new nuclei and therefore new starting points for fibrillation. As these terms stem from kinetic studies which measure the concentration dependence of different species in the aggregation process, little is known of the structural aspects of these mechanisms, although some studies have found structural clues (Scheidt et al., 2019; Gallardo et al., 2020). The kinetic profile of *in vitro* fibrillation starting from monomeric protein is described as a sigmoidal curve with two plateaus (Figure 2B):

(1) Lag phase: This phase comprises the time before fibrillation initiation until a sufficient mass of fibrils has accumulated for detection. While the first fibrils form rapidly, it takes some time until fibril mass has increased sufficiently by primary nucleation, elongation and secondary processes to become detectable. This leads to an apparent initial plateau (Arosio et al., 2015).
(2) Plateau phase: After the exponential growth phase an equilibrium is reached where the rates of fibril growth and disassembly are equal.

Kinetic parameters can be extracted from these curves *via* fitting to mechanistic models or empirical functions (Morris et al., 2009). One has to be careful with interpretation though, as the fibrillation process is very sensitive to experimental parameters and the presence of impurities. Linearity between the observed signal (e.g., the intensity of a fluorescent reporter) and the fibril concentration is also not a given. Therefore, only data sets acquired under the same conditions should be compared and analysis of a single curve is normally not pertinent (Ferrone, 1999). Nevertheless, with these precautions in place, a plentitude of information can be obtained from a fibrillation curve. Fitting to empirical functions such as the logistic function (Fink, 2006) is useful to compare different data sets, by measuring parameters such as lag time, half time, time to completion, maximal growth rate etc. However, interpretation of the so obtained parameters is not straightforward as regions in time- or parameter space cannot directly be related to one single microscopic parameter. In order to get a better insight in the microscopic process, fitting to mechanistic models is necessary. Multiple theories try to connect the macroscopically measured rates with microscopic processes. As for chemical kinetics, rate laws have been attempted to be established for aggregation processes starting in the 1960s, thanks to the work of Oosowa and subsequently Eaton, Ferrone and Hofrichter [Review: (Morris et al., 2009)]. Due to the multitude of processes and species involved and the resulting non-linearity of the mechanisms, this task is challenging. Nevertheless, integrated rate laws have been derived, which incorporate secondary pathways and depend only on a limited combination of the rate constants. Global fitting and analysis of singled out fibrillation pathways allows the extraction of these parameters (Cohen et al., 2011). Web-based software is now available, which allows global analysis of kinetic data without the need for extensive programming or detailed mathematical knowledge (Meisl et al., 2016).

**FIGURE 3:**
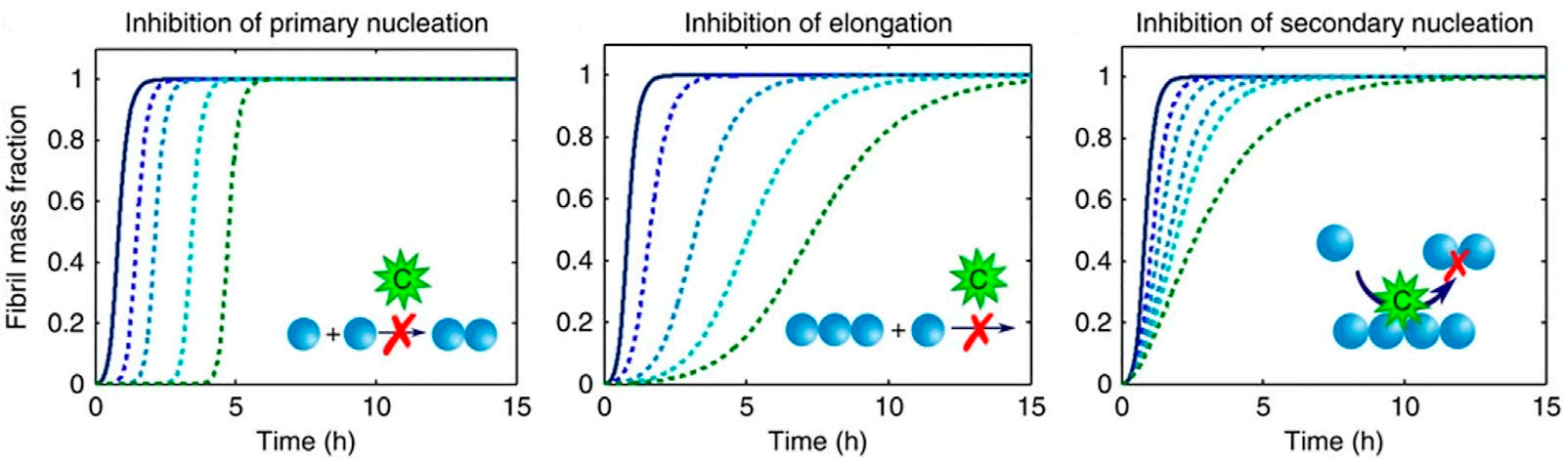
Inhibition of different molecular pathways modifies the observed fibrillation curves in characteristic ways (figure modified from Arosio et al., 2016). Simulation of perturbations of different microscopic pathways results in characteristic changes in the macroscopic kinetic profiles.

Importantly, fitting with mechanistic models allows studying the effect of inhibitors on the aggregation process (Figure 3). Inhibition of different microscopic mechanisms leads to distinct changes in the shape of the observed fibrillation curve. Thereby the effect of inhibitors on the fibrillation process can be determined. From knowledge of the inhibited process, the target species which interacts with the inhibitor can be found (Arosio et al., 2016; Michaels et al., 2020). This technique is used frequently in the study of interactions between amyloidogenic proteins and chaperones, as discussed further in Section 4.2.

## 3 Chaperones

Chaperones are a heterogeneous group of proteins, defined by their function as “helper-proteins”. Chaperones are able to prevent misfolding and aggregation, refold misfolded proteins, assemble complexes, disassemble or detoxify aggregates and target misfolded proteins for degradation. In order to achieve all these tasks, they cooperate with other members of the cellular environment. The mechanism by which chaperones interact with a multitude of misfolded species is distinct from other molecular machines and still an exciting field of active research (Burmann & Hiller, 2015; L. He & Hiller, 2019; Hiller & Burmann, 2018). They are natural allies against misfolding diseases and have become promising targets for pharmacological intervention by targeted upregulation or functional replacement (van der Putten & Lotz, 2013).

### 3.1 The chaperone family and its nomenclature

**FIGURE 4:**
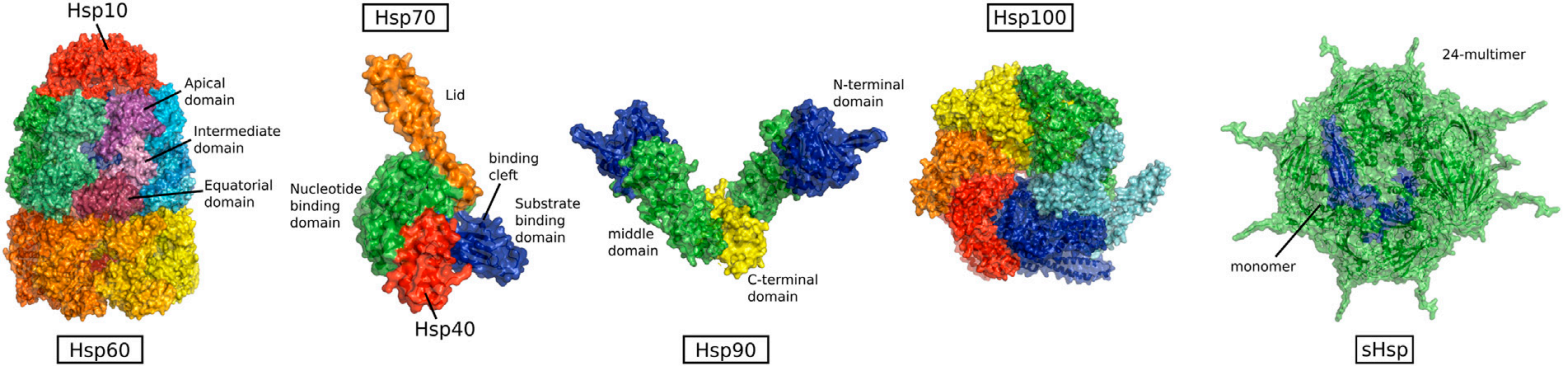
Structure of heat shock protein families, from left to right: HSP60 (PDB:1pcq, from GroEL-GroES E.*coli*) HSP70 in complex with HSP40 (PDB:5nro, DnaK and J-domain from E.*coli*), HSP90 (PDB:2ioq, HTPG from E.*coli*), HSP100 (PDB:5og1, ClpB from *E.coli*), and sHSPs (PDB:3J07, alpha-crystallin from human) (Not to scale).

The existence of chaperones was long unknown. Historically they have not been related to the normal functioning of the cell, but were first found in the context of stress, induced by heat shock (Ritossa, 1962). Because of this historical association, the main chaperones are called heat shock proteins (HSPs) (Figure 4) (Schlesinger et al., 1982). HSPs are among the most highly conserved proteins between different organisms and the major classes are found in all kingdoms of life (Lindquist & Craig, 1988). They are named by the size of their constituent subunits and can be grouped into the different families:

HSP100, HSP70, HSP90, HSP60, HSP40, and small HSPs (sHSPs).

However, they are known by a multitude of names, depending on the host organism or cellular location. Many studies have used bacterial and some archaeal model systems. They are also found in all compartments of the eukaryotic cell and therefore carry different names, even if from the same family. Besides this historical definition, chaperones were also defined as “any protein that interacts with, stabilizes or helps another protein to acquire its functionally active conformation without being present in its final structure”. This more inclusive definition encompasses proteins encoded by 332 genes (Balchin et al., 2016).

The substrates of chaperones are typically called “clients” and HSPs have a multitude of them as well as so-called co-chaperones, that interact with chaperones and with client proteins to increase specificity and functional properties. HSP90 has more than 20 co-chaperones determining its function (Li et al., 2012), and HSP70 is regulated by co-chaperones of the numerous HSP40 family as well as nucleotide exchange factors (NEFs). On the other hand, HSP60 mainly cooperates with one co-chaperone and HSP100 does not require many co-chaperones to process client proteins (Kim et al., 2013), but cooperates with Hsp70 (Rosenzweig et al., 2013; Mogk et al., 2015).

### 3.2 Chaperones in the cellular context

The first chaperones acting on newly formed proteins are bound to the ribosome and the nascent chain emerging from it. Interestingly, bacteria and eukaryotes have evolved different chaperones for this purpose. Trigger factor (TF) acts in bacteria, and ribosome-associated complex (RAC) together with NAC (nascent chain associated complex) acts in eukaryotes. The dimeric NAC complex protects the newly-nascent polypeptide and determines its sorting to different cellular components (e.g., endoplasmic reticulum for secretion) (Gamerdinger et al., 2019). The RAC complex is formed from a HSP40 homologue and HSP70 homologue (in mammals: MPP11 and HSP70LI) and recruits cytosolic HSP70, for further folding or translocation (Preissler & Deuerling, 2012).

Early cytosolic folding chaperones are HSP60 and HSP70 with their co-chaperones prefoldin or HSP10, HSP40 and NEFs (Nucleotide exchange factors), respectively. HSP60 has first been recognized for its role in tubulin and actin biogenesis (Yaffe et al., 1992), but has also been found to interact with a diverse set of other newly synthesized proteins (Thulasiraman et al., 1999). The most prevalent and most versatile chaperone is HSP70, at a cellular concentration of about 27 μM at 30°C in *E. coli* (double upon heat shock) (Mogk et al., 1999). Besides its interaction with NEFs and HSP40, HSP70 cooperates with sHSPs of which 10 homologues exist in mammals. sHSPs trap misfolded clients, until refolding by HSP70 is possible. The other major cytosolic chaperone, HSP90 is involved in late folding stages. It is functionally more specialized towards maturation of signaling proteins such as steroid hormone receptors and kinases (Saibil, 2013). A handover mechanism between HSP70 and HSP90 was reported, where the chaperone HOP is connecting HSP70 and HSP90 and thereby early and late folding pathways (Chen & Smith, 1998).

Transport of polypeptide chains into different cellular compartments of eukaryotic cells also requires chaperones. Peptides which are expressed on the cell surface or are secreted to the outside of the cell have to pass the endoplasmic reticulum (ER), where glycosylation and disulfide bond formation is happening in an environment high in Ca^2+^ (Michalak et al., 2002). Chaperones of the HSP90 family (Grp94) and HSP70 (Bip) with its HSP40 co-chaperones (ERDJ1-6) and NEFs are present in the ER, fulfilling similar roles as in the cytosol. HSP70 plays an important role in translocation, delivering and receiving proteins on the two sides of the membrane (Braakman & Bulleid, 2011). The majority of mitochondrial proteins are synthesized in the cytosol and have to be unfolded and subsequently refolded to be transported through the mitochondrial membrane. Therefore, mitochondria have a set of special intermembrane chaperones (Chacinska et al., 2009). Inside the mitochondria the most important chaperones belong to the HSP60 and HSP70 families, but also HSP90 (Trap1) and AAA + unfoldases (HsIU/ClpX, ClpA, ClpB) are found (Voos, 2013).

Also, in the extra-cellular fluid chaperones are found. Partly they are the same as cytosolic chaperones, but at lower concentration. This is however mostly related to tissue injury (Walsh et al., 2001). Due to the different physical conditions in interstitial fluid or blood, folding activity of chaperones is limited, as only very low levels of ATP are available (Gorman et al., 2007). Yet, there is a set of chaperones specific to the extra-cellular environment. They do not require ATP for their functioning, but rather keep the client proteins in an unfolded but unaggregated state. Clusterin, α-2-Macroglobulin (α_2_M), haptoglobin, and caseins are extra-cellular chaperones which have been found to have marked anti-aggregatory effects and are present in sufficient concentrations to be effective (Wyatt et al., 2013).

### 3.3 Chaperones in the proteostasis network

Proteostasis (protein homeostasis) describes the ability to control and balance concentration and type of proteins, the proteome, within a cell. Naturally, many processes play into this equilibrium, but typically the proteostasis network is defined by its immediate role in protein synthesis, folding, disaggregation and degradation, so it encompasses the translation machinery, molecular chaperones, the ubiquitin-proteasome system and the autophagy machinery (Labbadia & Morimoto, 2015). Chaperones are involved in folding and disaggregation, but also in degradation and autophagy. For example, the HSC70 co-chaperone complex interacts with the lysosomal membrane by recognition of a targeting motif, which leads to direct lysosomal membrane crossing and degradation (Massey et al., 2006). HSP70 and HSP90 also interact with the ubiquitin ligase CHIP to target proteins to the proteasome for degradation.

Mutations in proteostasis components have been found to relate to the early-onset of amyloid diseases, thereby providing a link between proteostasis and aggregation. For example, early-onset Parkinson’s disease is caused by loss-of-function mutations in the ubiquitin ligase PARKIN and its related kinase PINK1, which are involved in autophagy of mitochondria (Kitada et al., 1998; Leroy et al., 1998). Mutation of mitochondrial HSP60 leads to a neurodegenerative disorder with brain hypomyelination and leukodystrophy (Magen et al., 2008). Misfolding diseases are also related to old age, so they coincide with a decline of the proteostasis capacity during aging. However, the dependence between these two factors goes both ways. Age-dependent proteostasis decline has been shown to be the causal factor for amyloid disease onset (Douglas & Dillin, 2010), but it was also observed that protein aggregation leads to proteostasis impairment (Gidalevitz et al., 2006).

The connection between proteostasis capacity and misfolding diseases makes the proteostasis network a promising target for pharmacological intervention. Both chemical chaperones and upregulation of proteostasis components were shown to be beneficial for cells expressing aggregation-prone proteins and prevent the formation of toxic aggregates (Sittler et al., 2001; Chaudhuri & Paul, 2006; Hageman et al., 2010; Coelho et al., 2016). However, it was also shown that inhibition of integrated stress response pathways (and therefore downregulation of proteostasis components) improves cognitive impairment (Chou et al., 2017; Costa-Mattioli & Walter, 2020). This is related to the sensitivity of neurons on active translation for their functioning, which makes them especially vulnerable to aggregation-induced stress states and probably leads to the observed neural degeneration in protein misfolding diseases (Zhou et al., 2019). Therefore, a broad, constant upregulation of the whole proteostasis network by stress pathways is not a sustainable strategy for medical intervention. A better strategy is more targeted regulation and functional replacement by synthetic chaperones (Coelho et al., 2016), or upregulation of degradation pathways (Maiese, 2016). In order to fulfill these goals a detailed understanding of the working mechanisms of chaperones is necessary.

### 3.4 Chaperoning mechanism

Chaperones are so-called “molecular machines”. This means that certain triggers elicit movement, comparable to macroscopic machines (Ballardini et al., 2001). It is therefore important to consider both structure and dynamics of chaperones in order to understand their working mechanism. However, chaperones vary substantially from other molecular machines, such as enzymes, by their promiscuity and resultant low substrate specificity, therefore their types of interaction and mode of action are also accordingly different.

Three types of chaperoning activities are distinguished: folding, holding and disaggregating/unfolding. HSP60, HSP70 and HSP90 are foldases; they bind and release unfolded protein substrates fueled by ATP hydrolysis. This cycling mechanism leads to folding of the client protein. Chaperones without ATP hydrolysis capability are called holdases, they bind (hold) unfolded client proteins and thereby prevent their aggregation. HSP100, which can actively unfold misfolded aggregates falls under the category of disaggregases/unfoldases (Burmann & Hiller, 2015).

Two interaction modes are distinguished for chaperone-client complexes, according to their interaction energy landscape, determined by the conformational entropy of the bound client. In the single conformational limit, the client is predominantly bound in one conformation. This mode resembles classical protein-protein interactions and the interaction energy landscape has a narrow, steep valley for the single backbone conformation. There are for example protein-specific chaperones, e.g., HSP7 for collagen, which are able to bind the substrate after it is no longer recognizable by typical chaperones (Hendershot & Bulleid, 2000). The multi-conformational complex or fuzzy complex, is characterized by a multi-conformational ensemble of the client protein whilst it remains bound to the chaperone. The interaction between the bacterial chaperones Spy and Skp are examples of this (Burmann et al., 2013; He et al., 2016). This type of complex gives rise to a broad basin in the interaction landscape. Large conformational entropy adds favorably to the free energy of the interaction which is reflected by dissociation constants up to low μ-molar ranges (Hiller & Burmann, 2018).

An extended binding surface combined with dynamic binding ensembles allows for reorientation of the client protein whilst being bound to the chaperone. The concept of frustration refers to the conflicting penalties of different constraints of a system in its desire to reach a state of minimal energy, for example amino acid topology/connectivity and favorable interactions (Ferreiro et al., 2014). Folding intermediates are inherently in this state of conflict, which is also characteristic of their interactions with chaperones. Therefore, chaperone binding can increase backbone dynamics by sampling of multiple conformations in their search for an unfrustrated ensemble, which allows for a faster search for the right structure (L. He et al., 2016). This is an important part of the chaperone mechanism, as it allows backbone conformations to be explored by the client whilst intermolecular interactions are avoided (Sekhar et al., 2015). It is ultimately hydrophobic collapse which drives folding, so when folding occurs, previously available hydrophobic binding sites are buried, the client is released and further protein-chaperone interactions are discouraged.

Beyond shielding proteins from intermolecular interactions and waiting for folding to happen, chaperones can also be more active partners in the folding process. Compaction of the client protein encourages folding, because it destabilizes the unfolded extended state. This can be done by enclosing the protein chain in small spaces (Libich et al., 2017) or by subunit movements (L. He et al., 2016). Another important active function of chaperones is unfolding and disassembly of misfolded aggregates, processes which have been elucidated to astounding levels of detail. Typically, an ATP-fueled pulling mechanism is at their functional core (Gao et al., 2015; Maillard et al., 2011; Nachman et al., 2020; A. S. Wentink et al., 2020).

## 4 Interaction of amyloidogenic proteins with chaperones and inhibition of amyloid toxicity

Chaperones have been shown to intervene in the fibrillation process of amyloidogenic proteins, during multiple steps (A. Wentink et al., 2019). Many studies show that chaperones can inhibit aggregation, disaggregate or detoxify already formed amyloid fibrils. Additionally, in 35% of cases (Chiti & Dobson, 2017), amyloidogenic proteins are globular in their native state so classical refolding can be initiated by chaperones. One interesting aspect of interactions between chaperones and amyloidogenic proteins which will be discussed here is how chaperones recognize amyloid substrates. Also, the interaction of chaperones with different species and consequences for the fibrillation process will be presented before seminal studies of chaperones with amyloidogenic proteins are reviewed.

### 4.1 Substrate recognition

Other than typical protein interactions which are stabilized by two co-evolved binding surfaces with complementary polar-polar, charge-charge, and/or hydrophobic interactions, interactions of chaperones with misfolded client proteins do not have this complementarity. Binding is mostly mediated *via* interaction with exposed hydrophobic stretches which are typically exposed on unfolded proteins or folding intermediates (L. He et al., 2016). Multiple binding sites increase the affinity of chaperones to their substrates, as multiple low affinity sites add up to a higher binding affinity, which is referred to as avidity (Karagöz et al., 2014).

However, the amount of affinity has to be carefully balanced as too much affinity leads to an antifolding activity, as the residence time of the client is increased (Huang et al., 2016).

How does binding differ for the 65% of amyloidogenic proteins which are intrinsically disordered in their native state and therefore have different physicochemical properties than the typical chaperone client, the folding intermediate? Although intrinsically disordered proteins (IDPs) tend to be more hydrophilic overall, amyloidogenic IDPs contain hydrophobic stretches which subsequently form the *ß*-sheet core of the fibril. Without this feature, no amyloidogenicity is observed. Therefore, in this regard they partially resemble folding intermediates. Another important factor in binding is the availability of hydrophobic sites. Whilst globular proteins bury their hydrophobic residues, these stretches are constantly exposed on IDPs, which enables them to mediate intermolecular contacts. This increased availability of hydrophobic stretches explains how IDPs can resemble “normal” client proteins.

Besides these general principles, it has also been postulated that selective recognition motifs exist, which target certain amyloidogenic proteins, such as huntingtin (Monsellier et al., 2014) or Tau protein (Oroz et al., 2017) to HSP70 or HSP90. These motifs are rich in hydrophobic residues, but also in positively charged residues. Interestingly, they have a tendency towards α-helical structure which suggests that the interaction site might not be determined by primary, but by secondary structure elements (Hervás & Oroz, 2020). Burman and coll. find that six vastly different molecular chaperones commonly recognize a canonical motif in α-synuclein, consisting of the amino-terminus and a segment around Tyr39, hindering its aggregation (Burmann et al., 2020). DNAJB6/8 (HSP40) contains a region rich in threonines and serines, which is hypothesized to specifically interact with the *ß*-hairpin hydrogen bonding network of poly-Q proteins (Kakkar et al., 2016). The discovery of these specific binding motifs might suggest a co-evolution of amyloidogenic proteins with chaperones.

Chaperone action can also be directed towards fully formed aggregates for disassembly or detoxification. The factors determining binding to fibrils are lesser known and are thought to strongly depend on the type of fibril.

### 4.2 Functional and structural studies of HSPs and amyloidogenic proteins

For all major groups of HSPs structures are available, mainly from X-ray crystallography studies or since recently cryo-EM (Berman et al., 2000). However, insights into the dynamic mechanism of chaperones at work are not easily gained from static structures, yet this information is crucial as dynamics upon ATP binding, hydrolysis and ADP release play an important role in chaperone action, and also the client binding is often highly dynamic. Only few structures of chaperone client complexes have been elucidated so far, and all of them should be seen as structural ensembles rather than fixed complexes (L. He et al., 2016; Jiang et al., 2007; Mas et al., 2018; Weinhäupl et al., 2018). Methods such as targeted mutation or cross-linking MS (mass spectrometry) have given important insights on binding interfaces, but for chaperone studies, NMR (nuclear magnetic resonance) spectroscopy has established itself as the most powerful method due to its ability to investigate dynamic and unstructured systems (Hiller & Burmann, 2018). In the following, the mechanism of the main HSP-groups and important studies elucidating their interaction with amyloidogenic proteins will be presented.

#### 4.2.1 HSP70 and its co-chaperones HSP40(DNAJ) and NEF

**FIGURE 5:**
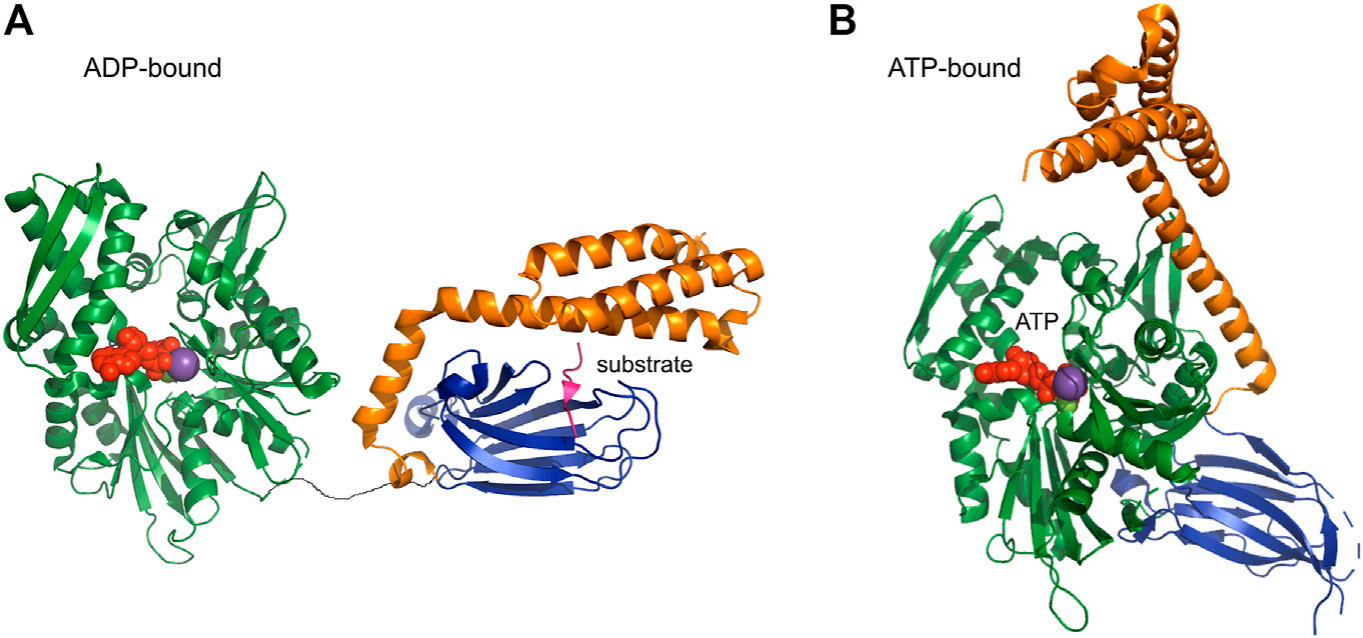
ADP and ATP-bound form of HSP70 [PDB codes: 2qxl (yeast), 4po2 (human)] HSP70 is composed of the nucleotide binding domain NBD (green) and the substrate binding domain SBD (blue), which are connected by a flexible linker. **(A)** In the ADP-bound form, NBD and SBD can move independently from each other. **(B)** Upon binding of ATP a large structural rearrangement occurs and the SBD and NBD bind together.

HSP70 is the most prevalent chaperone and is involved in all chaperoning processes such as folding, disaggregation, and translocation. Structurally it is divided into nucleotide binding domain (NBD) (in DnaK 44 kDa size) and substrate binding domain (SBD) (in DnaK 25 kDa size). The SBD has a *ß*-sandwich fold with a cleft for substrate binding and an α-helical lid. NBD and SBD domains are flexibly linked and have been elucidated by X-ray crystallography independently from each other. Solution NMR studies measuring RDCs (residual dipolar couplings) and relaxation showed that, in an ADP-bound state, the movements of the two domains are uncorrelated and that the linker is unstructured (Figure 5A). However, the relative motion of the subunits is restricted in a 35°C cone, that is, the motion of SBD with respect to NBD is restricted to a cone of about 70°C opening angle (Average structure: PDB: 2KHO) (Bertelsena et al., 2009).

ATP binding leads to attachment of both lid and SBD on NBD, thereby exposing the binding site, which is otherwise covered by the lid domain (Figure 5B) (Zhu et al., 1996). The attachment is not mediated by structural changes, as there are no structural modifications in the NBD upon binding of different nucleotides, suggesting a regulation by dynamics. ATP binding stabilizes a single state, whilst ADP bound NBD is in slow exchange between the “ATP-bound state” and another state (Revington et al., 2004). NEFs (nucleotide exchange factors) bind near the entrance to the nucleotide cleft, thereby opening it for increased nucleotide exchange (Harrison et al., 1997). The other class of HSP70 co-chaperones, namely HSP40 (or J proteins) binds on the flexible linker between NBD and SBD thereby exerting their regulatory function allosterically (Jiang et al., 2007).

HSP70 was found to be implicated at early steps in the fibrillation process, inhibiting the fibrillation of a multitude of amyloidogenic proteins (Aβ, α-synuclein, Huntingtin, IAPP) (Muchowski et al., 2000; Klucken et al., 2004; Evans et al., 2006; Monsellier et al., 2014; Burmann et al., 2020; Chilukoti et al., 2021). The majority of studies conclude that HSP70 is interacting with an oligomeric species. For example, Chilukoti et al. studied the interaction between IAPP and HSP70 in different solvent conditions. They found increased lag times and slower fibril growth upon addition of substoichiometric concentrations of HSP70 in ThT-fibrillation assays, even in the absence of ATP. The inhibition was found to be stronger when added at the beginning of the reaction and progressively less when added later. As measurement of the affinity between IAPP monomer and HSP70 was found to be weak, authors hypothesize the inhibition effect to be based on interaction between HSP70 and a rare intermediate oligomeric species (Chilukoti et al., 2021). The wealth of evidence suggests that a generalized effect of HSP70 in the fibrillation process is inhibition of primary nucleation by interaction with oligomeric species.

Two disaggregation mechanisms involving HSP70 have been described. One mechanism, involving HSP70 and disassembly by threading through HSP100, has been reported to happen in bacteria, plants and fungi. It involves delivery of unfolded protein to HSP104 or ClpB (HSP100) disaggregase by HSP70 (Rosenzweig et al., 2013) and will be discussed in the following section on HSP100.

The other mechanism of fibril disaggregation by HSP70 has been proposed recently (Gao et al., 2015; Nachman et al., 2020; A. S. Wentink et al., 2020). In 2015, Gao et al. described the kinetics of α-synuclein fibril disassembly by Hsc70 (constitutively expressed HSP70), together with the co-chaperones DNAJB1 and HSP110-type NEFs (not part of the HSP100 family). By combining EM, biochemical and fluorescence techniques they showed that individually none of the factors can disassemble fibrils, but HSP70, DNAJB1 and NEF together show a high disassembling efficiency. The follow-up study by Wentink et al. explained the underlying mechanism. NMR measurements were used to identify binding sites of the three partners (HSP70, NEF, DNAJB1) on monomeric α-synuclein. FRET (Förster resonance energy transfer) and truncation experiments were then used to validate the interaction regions on α-synuclein fibrils. Chaperone and co-chaperones all engage with disordered regions protruding from the fibril. Sets of FRET measurements were performed to determine the amount and density of HSP70 on the fibril as a function of different factors, e.g., the number of co-chaperones, and were correlated with efficiency of disaggregation. The group concluded that DNAJB1 and NEF lead to high density clustering of HSP70 molecules on the fibril surface, which leads to fibril disassembly by entropic pulling. That is, the steric clashes between HSP70 and NEF molecules resulting from this high-density arrangement lead to a fibril disassembly by excluded volume effects. Nachman et al. showed that the disaggregation effect of the HSP70-DNAJB1-NEF system can be extended to Tau fibrils. These studies allow a detailed insight into the mechanism of fibril disassembly by elucidating the physical forces acting at the microscopic scale.

Nachman and colleagues however also illustrate the potential negative effects of fibril disassembly by demonstrating the seeding capacity of resultant monomeric and oligomeric species. Although fibril disassembly seems intuitively like an effective mechanism against amyloid toxicity, it is not uncontroversial whether fibril disassembly is a protective or a toxic mechanism as it leads to an increase in aggregation-prone species (Tittelmeier et al., 2020). As an alternative to fibril dissagregation, detoxification of already formed amyloidogenic fibrils can be achieved by forming inert aggregates or by degradation. HSP70 is involved in degradation *via* its connection with HSP110 which is connected to the proteasome *via* the proteasome shuttle UBQLN2 (Hjerpe et al., 2016).

The HSP70 co-chaperone HSP40 in isolation is also able to interact with amyloidogenic proteins and inhibit their fibrillation. Mansson et al. (2018) and Österlund et al. (2020) studied the interaction between HSP40 (HSP70 co-chaperone) and Aβ. A motif of conserved S/T residues was found to be important for the interaction between HSP40 and oligomers. Mansson et al. described the decrease of Aβ fibrillation inhibition and binding to DNAJB6 with an increased number of S/T substitutions (Månsson et al., 2018; Österlund et al., 2020). Österlund and colleagues subsequently focused on primary nucleation and pre-nucleation oligomers of Aβ and the importance of conserved S/T residues on DNAJB6 for fibrillation inhibition. Kinetic analysis of Aβ fibrillation in the presence of WT DNAJB6 showed an effect on lag-phase but no effect on the growth rate, typical for inhibition of primary nucleation. Also inhibition at substoichiometric ratios of chaperone were observed, a sign for interaction between chaperone and oligomeric species. Addition of a DNAJB6 mutant where S/T residues were replaced by alanine showed no such effect, similar to cross-linked DNAJB6 (forcing oligomerization). The authors interpret these results based on an oligomeric model of DNAJB6 where the S/T residues responsible for binding are located at the subunit interface of an oligomeric state of DNAJB6. To link kinetic assays with structural insight, the authors performed native-MS on Aβ in presence and absence of DNAJB6 (WT and mutant). Only the presence of WT, not mutant DNAJB6 was able to reduce the amount of observed Aβ oligomers after a 1 h incubation period. Furthermore, analysis of the different charge-state distributions of Aβ oligomers and monomers present allowed to pinpoint which size and structural state of Aβ is targeted by DNAJB6. This study is an interesting example of investigation of the Aβ oligomer distribution and structural state in response to interaction with a HSP40 chaperone.

#### 4.2.2 Disaggregase and unfoldase HSP100

The disaggregase HSP100 is a unique type of chaperone which does not help to fold proteins, but unfolds them. HSP100 is a member of the AAA+ (ATPases Associated with diverse cellular Activities) protein family and is, for example, found as part of the proteasome, preparing proteins for proper degradation. Beyond that, the role of chaperones of this superfamily also includes disassembly of complexes or aggregates (Neuwald et al., 1999). Whilst the eukaryotic HSP100 is composed of subunits with a size of about 100kDa, its bacterial (ClpA, ClpB, ClpX) (Baker & Sauer, 2012) or archaeal (PAN) (Benaroudj & Goldberg, 2000) counterparts have differing subunit sizes, about 85 kDa, 95 kDa, 45 kDa, and 70 kDa, respectively.

Conserved is in all cases the hexameric ring structures with a narrow pore in the middle into which loops with hydrophobic and aromatic residues, especially tyrosines, protrude. This interface non-specifically binds protein chains. The long, dynamic N-terminus is targeted by cofactors and helps delivery to the central substrate binding site (Cranz-Mileva et al., 2008). ATP-hydrolysis fuels rotation of the AAA + domains which pulls at the attached chain. Mechanical unfolding ultimately involves crossing of an energy barrier; by constant pulling HSP100 decreases the magnitude of this barrier, while increasing the chance that eventually a thermal fluctuation within the protein substrate will allow its crossing (Maillard et al., 2011). This power stroke mechanism allows HSP100, to disaggregate extremely stable misfolded proteins, such as amyloid fibrils (Motohashi et al., 1999). A review of disaggregation machineries can be found in (Mogk et al., 2018).

A detailed study of DnaK (HSP70) and ClpB (HSP100) has elucidated the molecular mechanism of fibril disaggregation on a set of non-amyloidogenic model proteins. Rosenzweig et al. (2013) show a NMR–derived structure of the ClpB-DnaK complex, calculated from chemical shift perturbations and PRE-restraints (paramagnetic resonance effect) and verified through mutagenesis and functional assays. The authors found that ClpB interacts selectively with the nucleotide binding domain of DnaK. Interaction is mediated between a coiled-coil propeller, located near the first ATPase domain on HSP100, and the nucleotide binding domain of HSP70. HSP70 exposes ends of substrates on which HSP100 can subsequently pull, increases the activity of HSP100 and binds the newly disaggregated proteins.

Although the disaggregation activity of Hsp100 would make it a useful medical target for amyloid disaggregation, structural/mechanistic studies which focused on Hsp100s effect on amyloid aggregates are few and far between. The size of the oligomeric AAA + ATPase, complexity of aggregated biological substrates, structural rearrangements triggered by ATP binding and hydrolysis, and the intrinsic low stability of several HSP100s, all present an array of logistical problems. Therefore, only few published studies reporting how HSP100 processes aggregates and amyloid are available. A notable exemption of this is the study of De Santis and colleagues. They studied yeast HSP104 and the dependence of its amyloid disaggregation mechanism on inter-subunit co-operation. The authors found the disaggregation mechanism of yeast HSP104 to differ depending on the type of aggregate (disordered aggregates vs. amyloid) and show the importance of inter-subunit co-operation. By mixing WT subunits with mutants defective in ATP hydrolysis or substrate binding the effect of cooperativity was investigated. Varying the amounts of mutant subunits and monitoring the disaggregation ability of the resultant chaperones towards amyloid and disordered aggregates allowed to conclude if the collaboration amongst subunits is probabilistic, subglobal co-operative or co-operative. The group found that in order to disaggregate disordered aggregates HSP104 subunits work independently from each other and are tolerant to dysfunctional subunits. Interestingly, they show that HSP104 works different from the related ClpB and ClpX which are dependent on co-operative ATP-hydrolysis. In order to disassemble amyloid fibrils a subglobal co-operative or co-operative mode is used by HSP104, dependent on the stability of the amyloid (Desantis et al., 2012). This study highlights the fundamental difference between disassembly of disordered aggregates and amyloid. In order to understand the implications on the molecular level more detailed structural studies of this process will be necessary.

#### 4.2.3 sHSPs

sHSPs are small, ATP-independent holdase chaperones, which assemble to “molecular cages” of differing sizes. These oligomers bind unfolded proteins and thereby prevent their aggregation, without actively folding them. For this purpose, they recruit HSP70, although apparently without direct interaction between the two chaperones. When unfolded proteins largely outnumber sHSPs, big aggregate structures, such as inclusion bodies are formed. However, other than normal aggregates, the protein aggregates in presence of sHSPs can be more easily disassembled and protein retrieved (Specht et al., 2011).

**FIGURE 6:**
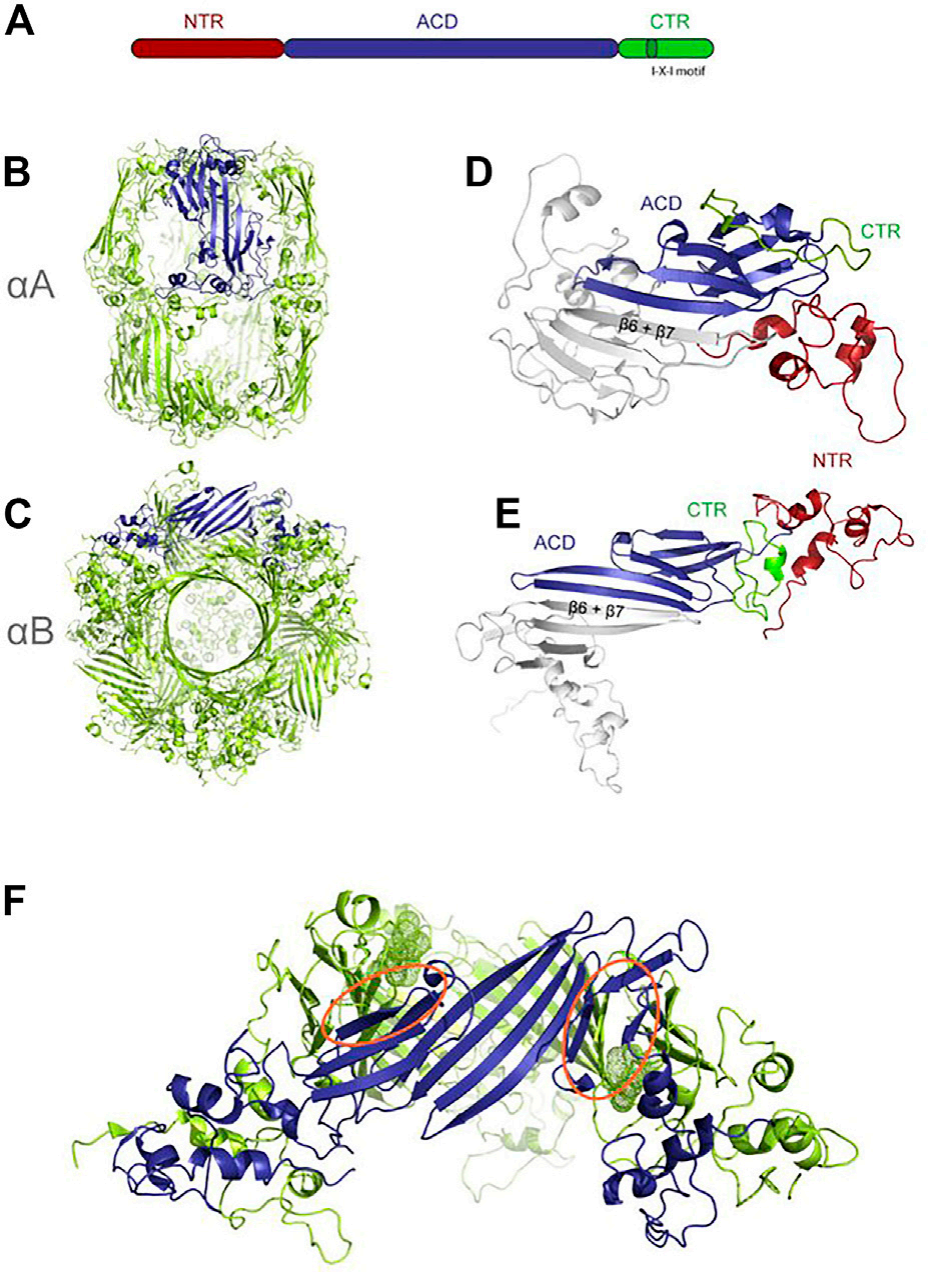
Structure of sHSPs, re-printed from (Riedl et al., 2020) **(A)** different domains of sHSPs **(B)** aA (PDB: 6T1R) **(C)** aB (PDB:2ygd) one monomer inside the oligomer is colored in blue for emphasis **(D,E)** dimers extracted from **(B,C)**, one molecule colored according to domains. **(F)** hexameric subunit extracted from aB with the interacting IXI motifs highlighted as dots.

Monomers of sHSPs (12 kDa–43 kDa) contain three domains, a flexible N-terminal domain, an α-crystallin middle domain (ACD), and a short C-terminal domain, containing the IX (I/V) motif (Figure 6A). No crystal structures exist of the very long (up to 250 aa) N-terminus (Haslbeck et al., 2004), but structural propensities have been measured and suggest a dynamic structural ensemble (Jehle et al., 2011). In mammals, the N-terminus is target of regulative phosphorylation. The 90 aa–100 aa long α-crystallin domain has a *ß*-sandwich fold, formed by seven *ß*-strands arranged in two antiparallel sheets and is the defining motif in this large family. The IX (I/V) motif in the C-terminal domain consists of three conserved amino acids and is important for oligomerization.

Oligomer size varies between 12- to 32-mers, typically containing even numbers of monomers as the structural unit is a homodimer formed by the ACDs. However, oligomers as small as dimers are reported. ACD interaction is very weak, and requires “cross-linking” with the IX (I/V) motif on the C-terminus binding to adjacent ACDs and dynamic N-terminal interaction. Both the 3 residue IX (I/V) motifs in the C-terminus and the N-terminus are important for oligomerization (Figures 6B–F) (Delbecq & Klevit, 2013).

sHSPs bind a multitude of clients, although it is not yet clear how substrate recognition happens. The N-terminus is enriched in hydrophobic residues and is therefore thought to be predominantly involved in substrate binding, but cross-linking studies coupled with MS showed interaction of client proteins with all sHSP domains (Åhrman et al., 2007). Studies by NMR spectroscopy showed that the Aβ-peptide is predominantly bound by the ACD, whereas unfolded lysozyme binds to the N-terminal domain (Mainz et al., 2015). Selig et al. studied the roles of C- and N-terminal domains and central α-crystallin domain of the two sHSPs human αB-crystallin (HSPB5) and HSP27 for binding of amyloidogenic proteins and fibrillation inhibition. The authors examined the effect on α-synuclein and apolipoprotein fibrillation using fibrillation assays and sucrose sedimentation. They tested different constructs with and without the terminal regions and assessed their effects on *in vitro* fibrillation inhibition. Although all the tested constructs, besides isolated α-crystallin domain, had some chaperoning function, they found the N-terminal region of HSP27 and both terminal regions of HSPB5 to be important in order to observe an increase in lag-time. Sucrose pelleting assays with subsequent SDS-PAGE were used both to assess the fraction of remaining monomers after fibrillation and assess binding between chaperone constructs and amyloidogenic proteins. Consistent with the fibrillation assays, all chaperone constructs which showed inhibition were found co-pelleted and therefore co-localized with fibrils. Furthermore, the authors showed an increase in the amount of soluble monomers resulting from apolipoprotein fibrils incubated with HSPB5, an effect which could not be reproduced with α-synuclein fibrils. Lastly, SV-AUC (analytical ultracentrifugation) and light-scattering experiments showed an increase in aggregate weight in the presence of full-length chaperone, which was interpreted as lateral association between the fibrils (Selig et al., 2020). This study confirmed the importance of the N-terminal region of sHSPs. However, for other substrates the binding site might differ, therefore more substrates need to be evaluated in future studies. This will allow to determine if a trend for substrate-binding on sHSPs exists dependent on the biophysical properties of the client proteins.

It had been previously reported that oligomeric state is important for sHPS’s action, with smaller oligomers having been observed as more active. It was postulated that the presence of stressors or unfolded clients can induce oligomer disassembly and thereby the activation of sHSPs (Sudnitsyna et al., 2015). Scheidt et al. (2021) studied the interaction between α-synuclein fibrils and αB-crystallin utilizing a microfluidic platform. They found marginal binding of the chaperone to α-synuclein monomers whilst fibrils are strongly bound. Therefore, they further quantified thermodynamics and kinetics of binding to α-synuclein fibrils. The ratio of α-synuclein (in the fibril) to αB-crystallin was determined to be 5.4 and a nanomolar affinity was found. Enthalpic and entropic contributions were calculated by non-linear van’t Hoff analysis; binding is strongly entropy driven, most probably brought about by a disassembly of oligomeric chaperones, as concluded from a positive change in heat capacity which excludes a hydrophobic effect. Quantification of the thermodynamic and kinetic parameters associated with the binding of αB-c to α-synuclein fibrils provides important information on the mechanisms through which sHSPs are able to sense misfolded protein aggregates and how disassembly to a more active chaperone-form is triggered by aggregates.

#### 4.2.4 HSP90

**FIGURE 7:**
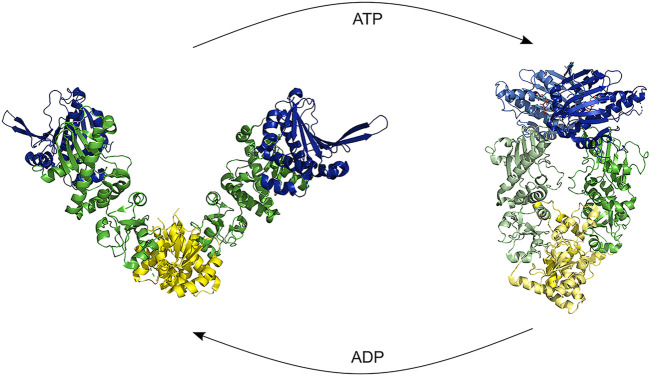
HSP90 opening and closure. Binding of ATP shifts the conformation from an open structure to a closed structure by dimerization of the NTD. Hydrolysis causes first a compaction of the proteins and a subsequent ADP release restarts the cycle [Structures: 5uls (dog), 2ioq (E.*coli*)].

HSP90 is an abundant cellular chaperone which acts at late stages of folding and on a more limited set of substrates as compared to HSP70. It is a dimer, with each subunit created from three flexibly linked domains: N-terminal, middle, and C-terminal domain. The subunits are stably connected *via* the C-terminal domain, creating a V-shaped molecule. It is however not fixed in its conformation, but dynamically sampling open and closed conformations (Figure 7) (Krukenberg et al., 2011). ATP binding to the N-terminal domain causes binding between the N-terminal domains *via* extensive inter-domain and inter-strand interactions, thereby stabilizing the closed conformation and shifting the conformational equilibrium towards the closed state (Ali et al., 2006).

The many binding partners of HSP90s, such as co-chaperones and client proteins, modulate its functional state. Also, post-translational modifications such as phosphorylation and acetylation modulate HSP90 activity (Pratt & Toft, 1997). One of many co-chaperones is the HSC70-HSP90-organizing protein (HOP). This protein binds with its TPR (tetratricopeptide repeat) domain motifs which are found both on HSP70 and HSP90 C-terminus, creating a complex between them for substrate handover (Chen & Smith, 1998). Little is known about HSP90 client complexes and its chaperoning function. It seems as if all three domains of HSP90 are involved in substrate binding (Saibil, 2013; Hiller & Burmann, 2018) and it has been reported that the fast dynamics of HSP90 are slowed down upon client binding (Lorenz et al., 2014).

Similar to HSP70, HSP90 has been found to inhibit aggregation of a wide array of substrate proteins (α-synuclein, Aβ, huntingtin, Tau) (Burmann et al., 2020; Evans et al., 2006; W. T. He et al., 2017; Karagöz et al., 2014). Especially interesting is the involvement of HSP90 in Alzheimer’s disease. It was not only found to modulate Aβ and Tau protein, but can also chaperone the kinases which phosphorylate Tau (Bohush et al., 2019).

Two in-depth structural studies of HSP90 interaction with Tau elucidated important mechanistic information by integrating NMR spectroscopy with other structural techniques, modeling and functional studies (Karagöz et al., 2014; Oroz et al., 2018).

Karagöz et al. (2014) combined NMR studies with small-angle X-ray scattering (SAXS). By titration the group determined the affinity of HSP90 to Tau to be in the low micro-molar range and determined the binding region on Tau by NMR titration. The binding interface contains the whole microtubule-binding region and is characterized by the combination of a net positive charge and the abundance of large hydrophobic and aromatic sidechains. It is larger, although partially overlapping, than a previously reported HSP70 interaction site. The reverse titration found a large binding interface of 840 Å^2^ on HSP90 which covers both its middle- and N-terminal domain. Addition of ATP did not change the affinity between Tau and HSP90, but modulated signal intensities on HSP90, which suggests an importance of dynamics in the chaperoning process. To obtain the overall shape of the complex, SAXS experiments were conducted. The group concluded that neither ATP binding, nor Tau binding changes the overall shape of HSP90 in solution and calculated interaction models utilizing the SA XS and NMR constraints. Furthermore, they analyzed the physicochemical characteristics of the HSP90 binding interface in depth and hypothesized that the scattered nature of hydrophobic stretches on Tau resembles typical chaperone clients, folding intermediates.

Oroz et al. (2017) studied the complex of HSP90 with Tau in presence of the co-chaperone PPIase FKBP51, which was previously reported to promote amorphous aggregation and neuronal death. Using an extensive integrative approach, the group determined the dynamic complex of HSP90/FKBP51 and HSP90/FKBP51/Tau and propose a mechanism in which the conformation of the ternary complex leads to a positioning of Tau’s proline rich region next to the catalytic pocket of FKBP51.

These studies allowed unprecedented high-resolution structural insight into the complex of HSP90 with an amyloidogenic client protein and explain how the interaction is modulated by one of HSP90s many co-chaperones. Structural insight into chaperone/co-chaperone interaction is especially useful as it could allow to tailor potential drug development towards inhibition of amyloid formation without disrupting the ubiquitous other functions of HSP90.

#### 4.2.5 HSP60 or chaperonin

HSP60s, also known as chaperonins, act at early stages of folding (Saibil, 2013). They are universal ATP-dependent foldases, found in all kingdoms of life. Chaperonins are assemblies of 14–18 subunits, which makes them big molecular machines of about 1 MDa in total. They are composed of two rings, depending on the organism formed by 7, 8, or 9 subunits each, placed back-to-back. For example, cytosolic chaperonin containing TCP1 (CCT), the eukaryotic cytosolic chaperonin, is composed of eight different subunits, whereas eubacterial chaperonin type I is composed of seven similar subunits and archaeal chaperonins (thermosomes) are composed of octameric or non-americ rings from one, two, or three different subunits (Yébenes et al., 2011). Each ring encloses a cavity in which protein folding takes place. Every subunit is divided in equatorial, middle and apical domain. The equatorial domains form the double-ring structure and contain the ATP binding side. The apical domain is responsible for client attraction and ATP-dependent cavity closure, mediated by an allosteric mechanism involving the middle domain (Yébenes et al., 2011).

This family is divided into two types, depending on their localization in the cell and structural characteristics. Type I is located in eukaryotic organelles as well as bacteria and type II in the eukaryotic cytosol and archaea. Group II chaperonins, having an additional helical protrusion to close their folding chamber, do not need a co-chaperonin for their closure mechanism, in contrast to group I chaperonins, requiring co-chaperonin HSP10 to close. Nevertheless, group II chaperonins are assisted in the protein folding mechanism by the co-chaperonin prefoldin (PFD), which functions as a holdase and transfers unfolded protein into the chaperonin cavity (Yébenes et al., 2011).

Chaperonins have a functional cycle of cavity opening and closing, mediated by ATP binding, hydrolysis, and ADP release. Some groups have proposed that ATP binding is sufficient for cavity closure (Nakagawa et al., 2014), and others that both binding and ATP hydrolysis is necessary (Reissmann et al., 2007). A study on thermosome (archaeal HSP60) conducted in our laboratory found that ATP bound and closed states coincide and that binding of non-hydrolyzable ATP closes the cavity, concluding that ATP binding is sufficient for closure (Mas et al., 2018). Intra-ring positive cooperativity and inter-ring negative cooperativity between the two rings in this mechanism is the consensus (Horovitz & Willison, 2005). However also a non-concerted mechanism has been observed for type II chaperonins (Bigotti et al., 2006). Cavity closure is of high importance for the chaperoning mechanism. Substrate is bound by exposed hydrophobic residues in the apical domain and equatorial domains near the sensor loop (Muñoz et al., 2011). Upon closing, the surface changes and hydrophilic, negatively charged residues are exposed, releasing bound substrate inside the cavity. It is proposed that restriction of conformational space and protection from intermolecular interaction constitute the folding mechanism of chaperonins (Brinker et al., 2001; Tang et al., 2006). This mechanism seems to be the case for both type I and type II, without any observable conservation in the binding interface and independent from ATP-driven cycling rates.

Sot et al. (2017) studied the interaction of HSP60 with α-synuclein. They performed fibrillation assays in the presence of ATP and ADP and observed substoichiometric fibrillation inhibition. TEM imaging of the resultant aggregates showed that interaction resulted in majoritively amorphous aggregates. By deletion studies, the central hydrophobic NAC region of α-synuclein was found to be important for interaction with HSP60. Cross-linking experiments and TEM imaging allowed to located two subunits as the interaction site on HSP60. Beyond this mechanistic and structural work this extensive study also tested cytotoxicity in cell cultures.

A lot of studies using a variety of biophysical techniques were performed on the interaction of HSP60 with Aβ (Mangione et al., 2016; Ricci et al., 2019; Vilasi et al., 2019). Mangione et al. (2016) studied the interaction of (mitochondrial) HSP60 with Aβ. They performed fibrillation assays and tested the structure of resultant aggregates by circular dichroism, AFM, TEM, and SEC and came to the conclusion that HSP60 selectively interacts with small oligomers. Vilasi et al. (2019) studied interaction of HSP60 with Aβ. Dose-dependence analysis of fibrillation assays showed HSP60 inhibition of nucleation processes. SAXS curves recorded as a function of time show a difference in the size and form of resultant aggregates in the presence of HSP60. Ricci et al., 2019 used neutron scattering to study the impact of Aβ on membranes in absence and presence of HSP60. Consistent with other studies they found that the presence of Aβ increased membrane stiffness, but it remains unaltered when HSP60 is present too.

**FIGURE 8:**
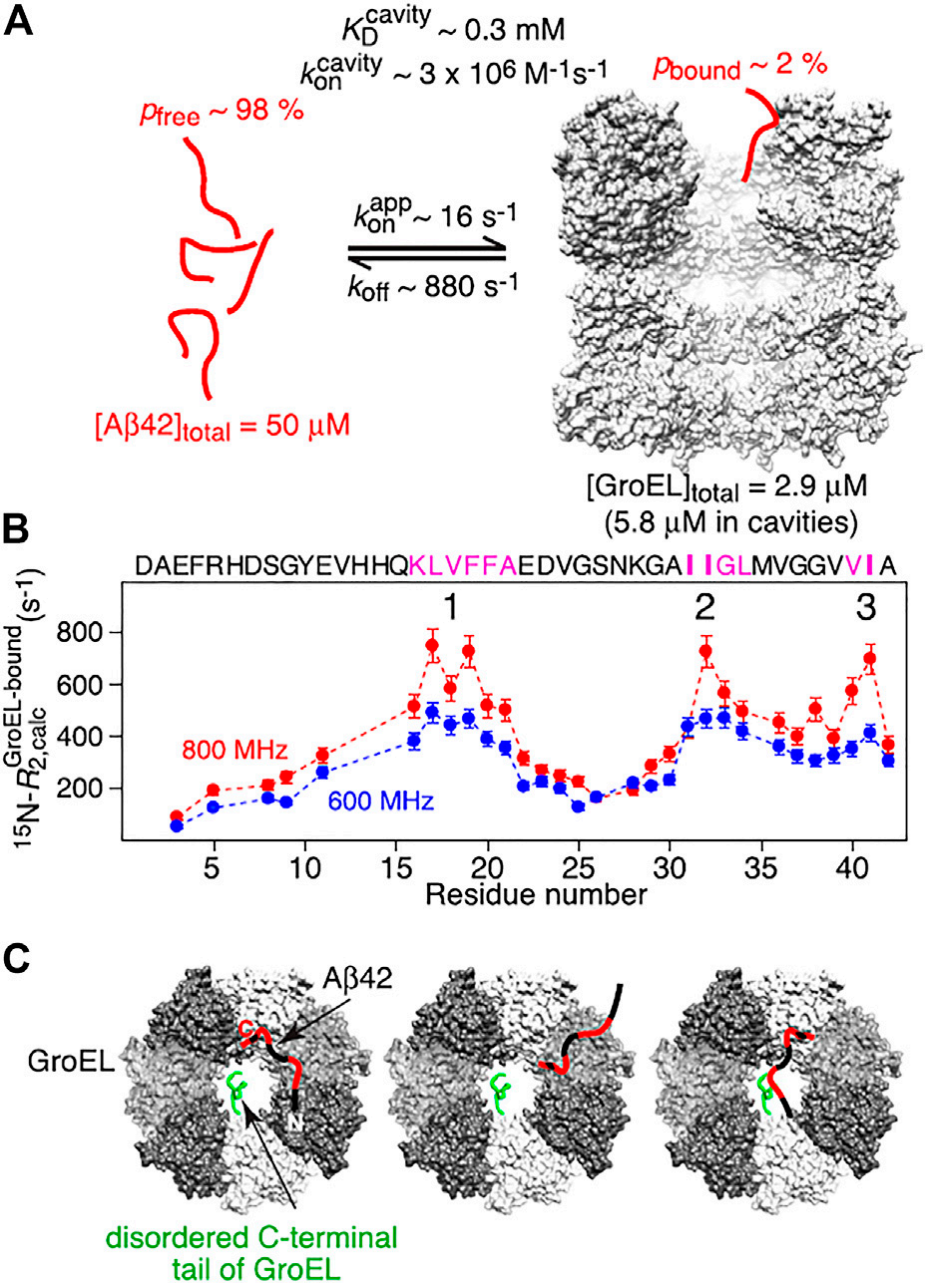
Summary of NMR-based analysis of Abeta/Hsp60 complex. Reprinted from Wälti et al., 2018 with permission. Copyright 2018 from National Academy of Sciences. **(A)** Kinetic parameters obtained from global best fitting of NMR relaxation experiments. **(B)** the three interaction regions in pink show the highest R2 values when bound to GroEL. **(C)** Schemes of potential interaction models which interconvert on a timescale shorter than the lifetime of the complex. Primarily interacting residues are colored in red, the different GroEL subunits are colored in gray scale and one disordered C-terminal tail of GroEL is depicted in green on one subunit.

Wälti et al. (2018) also studied the interaction between HSP60 (GroEL) with Aβ and obtained extensive structural and functional insights. Cell assays showed a decrease in toxicity of Aβ aggregates in the presence of HSP60. It was shown by time-resolved NMR experiments that HSP60 inhibits fibrillation as the disappearance of Aβ signal is slowed down in its presence. Dynamic light scattering even indicated that HSP60 either binds or disassembles small aggregates as the associated scattering intensity is decreased. Besides the mechanistic investigation, in this extensive study the interaction between HSP60 and Aβ was characterized in depth. Kinetics of the complex was investigated by 15N transverse relaxation rates, extracted from a combined analysis of 15N-lifetime line broadening (ΔR2), dark state exchange saturation transfer (DEST), exchange-induced chemical shifts, and Carr–Purcell–Meiboom–Gill (CPMG) relaxation dispersion. From these studies the lifetime of the complex was determined to be about 1 ms and three binding hotspots were determined on Aβ (Figure 8). Single molecule FRET analysis of doubly labeled Aβ showed that Aβ remains unfolded when bound to HSP60 (Wälti et al., 2018).

**FIGURE 9:**
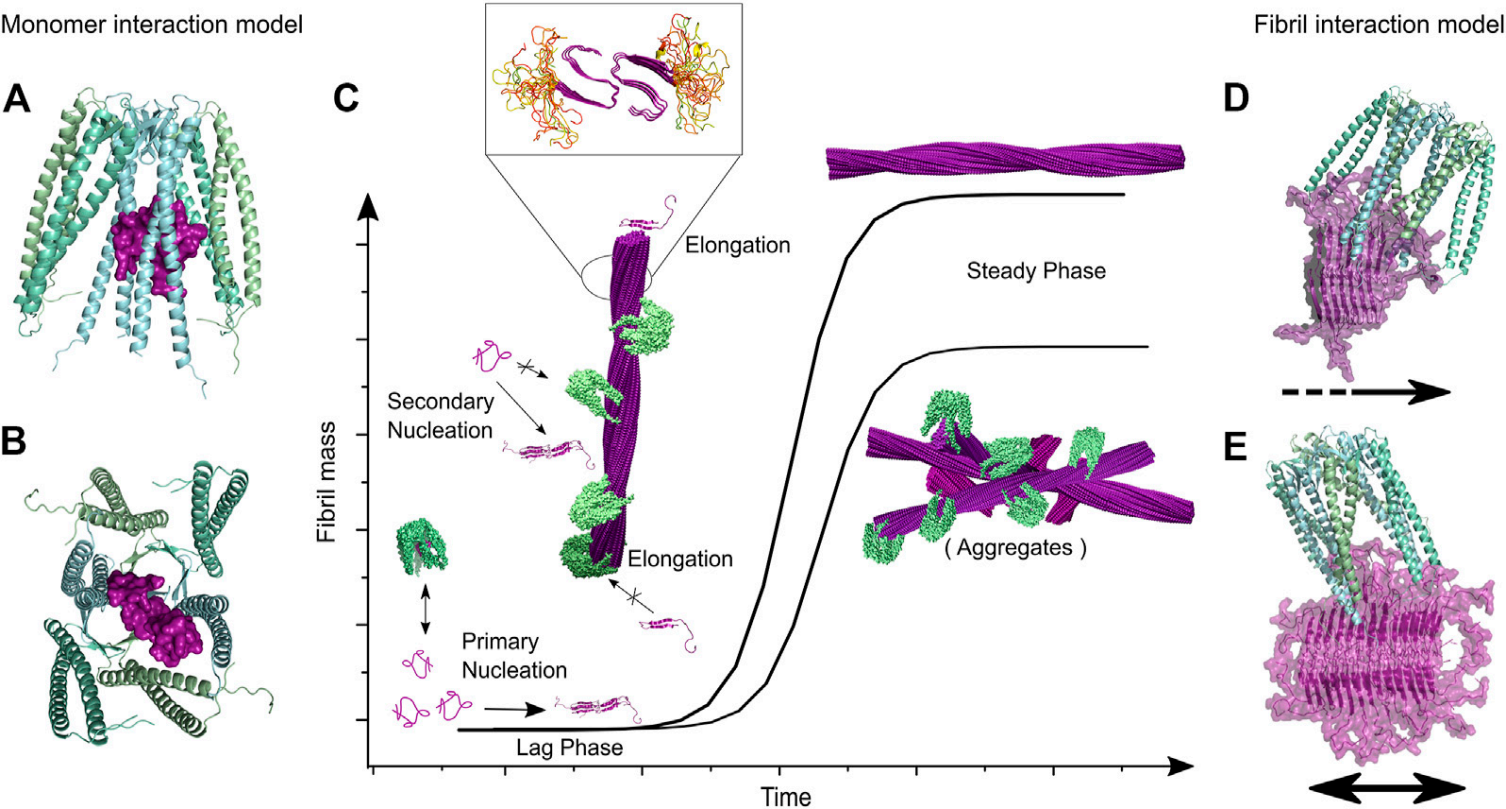
By combination of biophysical methods, microscopy and NMR spectroscopy Törner and coll. have elucidated the fibril inhibition of islet amyloid polypeptide by co-chaperonin prefoldin (reprinted from Törner et al., 2022). **(A,B)** present docking models of the complex between monomeric IAPP and PhPFD based on NMR derived interaction restraints. It was found that PFD interacts with monomeric IAPP, but this transient interaction does not lead to a significant decrease of the lag-phase. The major inhibition effect results from inhibition of secondary nucleation and elongation by interaction of PFD with fibril ends and surface. **(C)** Coverage of fibril surface and ends by PFD as found by negative stain EM. The presence of PFD leads to a decreased steady phase fibril mass, as seen in Tht-fibrillation assays, which results from the formation of less aggregates with an altered morphology, as shown by AFM. The inset zoom represents a model of IAPP fibril structure with unfolded residues 1–12 in yellow and the structured fibril core represented in purple (from residues 13–37). **(D,E)** present docking models of PhPFD on IAPP fibril surface and ends, respectively, integrating structural information obtained by NMR and EM.

A study performed in our group focused on the co-chaperonin of HSP60, prefoldin (PFD). Fibrillation assays and a combination of AFM, EM and NMR spectroscopy allowed to explain the species involved in prefoldin’s inhibition of the IAPP (islet amyloid polypeptide) fibrillation process and determine the structural basis for inhibition (Figure 9). Fibrillation assays showed substoichiometric inhibition, due to inhibition of elongation and secondary nucleation. A change in aggregate form suggested by the fibrillation assays was investigated by AFM imaging, which showed formation of clustered aggregates, which probably explains the toxicity-reducing effect observed in cell-assays. Combination of electron microscopy of fibrils in presence of prefoldin and NMR restraints (CSPs and PRE-data) allowed to calculate models of the fibril-prefoldin interaction and suggested that inhibition is mainly due to interaction of prefoldin with fibril ends and surfaces, attached to protruding N-terminal residues of IAPP (Törner et al., 2022).

Interestingly, in these studies both the HSP60 chaperonin and its co-chaperonin prefoldin have been reported to interact with amyloidogenic proteins with hydrophobic residues located in (co-)chaperonin cavities. While the interactions with monomeric amyloidogenic proteins are weak, stronger interactions were observed between small oligomeric intermediates and chaperonin or with the surface of larger fibrils and (co-) chaperonin (Wälti et al., 2017; Törner et al., 2022) The interaction with larger species seems to be the main contribution to the inhibition of fibril formation by reducing concentration of free oligomers, or by blocking fibril elongations (co-)chaperonin bound to fibril tips and secondary nucleation (co-)chaperonin bound to fibril lateral surfaces. Sequestration of toxic oligomeric species, reduction of the amount of aggregate and formation of bigger size aggregates composed of shorter fibrils clustered together have also been proposed to contribute to the detoxification mechanisms.

## 5 Discussion and future developments

Amyloid diseases remain an unsolved threat to our society to this day; many promising drug candidates have failed to cure the associated neurological or systemic diseases. Harnessing the proteostatic network, which is the natural defense mechanism against misfolding diseases, is a promising alternative strategy. In particular chaperones, which can modulate the conformational state of their client proteins, can be harnessed for this endeavor. The field of chaperone–amyloidogenic protein studies has been increasing in the last 20 years and has produced a wealth of information. Insights into fibril structures and even tentative structures of oligomeric intermediates have been elucidated. Important theories regarding the toxicity of amyloid have been developed and the interconnectivity of the proteostasis network and functional understanding of the class of chaperones has been achieved.

The field of chaperone-amyloidogenic protein interactions has developed and employed an impressive amount of experimental and computational techniques. Kinetic investigation of the fibrillation process has reached a level of maturity which allows the application of mechanistic fitting to fibrillation curves with powerful, widely available software. The standard application of in-depth analysis of different fibrillation pathways and hence the associated species will allow further interesting insights in the future. Important biophysical techniques to investigate amyloidogenic proteins are circular dichroism spectroscopy, dynamic light scattering and FRET. The combination of biophysical techniques with structural biology techniques has been shown to give the most impressive insights into the mechanisms of interaction between chaperones and amyloidogenic proteins. Atomic force microscopy, electron microscopy, cross-linking mass spectrometry and NMR have been used to get insights into fibril structures, interactions between chaperones and amyloidogenic proteins and their complexes. NMR has established itself as leading structural technique for the study of chaperones; its utility for application to chaperone-amyloidogenic interactions is equally clear. Investigation of dynamics on chaperone and amyloidogenic clients by NMR spectroscopy allows to shed light on the biochemical and biophysical mechanisms.

Despite the experimental and technical advances, our understanding of the associated processes needs to gain in depth in order to functionally replace chaperones or selectively upregulate them for combating amyloid toxicity. The most urgent open question relates to the nature of the decisive toxic aggregate species. The spreading of amyloid fibrils over the affected organ has been linked to disease progression. However, neurodegeneration seems to be caused by smaller oligomeric species rather than by elongated amyloid fibrils. This argues against the applicability of fibril dissociating chaperones or chaperone-like molecules as therapeutic candidates for amyloid diseases, since they can increase the number of seeding-competent and possibly toxic oligomeric species. Along these lines, a valid strategy is thought to be the reduction of oligomers of amyloidogenic proteins. The network of heat shock proteins offers several options to act in this direction: HSPs may i) interfere with the formation of critical oligomeric species from monomeric precursors, ii) remodel toxic oligomers into benign aggregates, iii) serve as targets to promote fibril elongation and to disfavor fibril dissociation into oligomers, iv) initiate oligomer degradation. The studies reviewed above provide evidence for all these HSP activities. As HSPs target client multimers, they typically affect amyloid assembly at substoichiometric concentrations. A further interesting feature of HSP interactions, that has also been noted for amyloidogenic targets, is their limited sequence specificity. This may allow to transfer a chaperone-based therapeutic strategy to a whole range of protein misfolding diseases. However, before such strategies can emerge, critical challenges have to be addressed. Most importantly, chaperone mechanisms as well as protein aggregation are complex sequences of reactions involving a plethora of protein states at diverse locations in the affected organ. Despite these challenges, studies of the interaction of HSPs with amyloidogenic targets have already provided exciting and detailed insight into the formation and properties of amyloid assemblies and their modulation. With the established experimental toolbox, we expect an influx of insightful studies to come out in the upcoming years, giving a more and more detailed view on how the interaction of chaperons with amyloidogenic substrates leads to the prevention of amyloid toxicity. These studies will hopefully prepare the way for future medical breakthroughs and help to treat notoriously difficult amyloid diseases.
